# Supplementary material for: Mitochondrial protein BNIP3 regulates Chikungunya virus replication in the early stages of infection
Source: PLoS Negl Trop Dis. 2023 Nov 27;17(11):e0010751. doi: 10.1371/journal.pntd.0010751 (PMC10703415; doi:10.1371/journal.pntd.0010751)
Supplement: S1 Fig — (A) Percentage of GFP+ (infected) cells after each time-point. (B) Bar plot showing the signal-to-noise ratio after each time point, i.e, the mean fluorescence intensity (MFI) from infected wells (signal) divided by the MFI of the corresponding mock (noise). (DOCX) [file pntd.0010751.s001.docx]

**
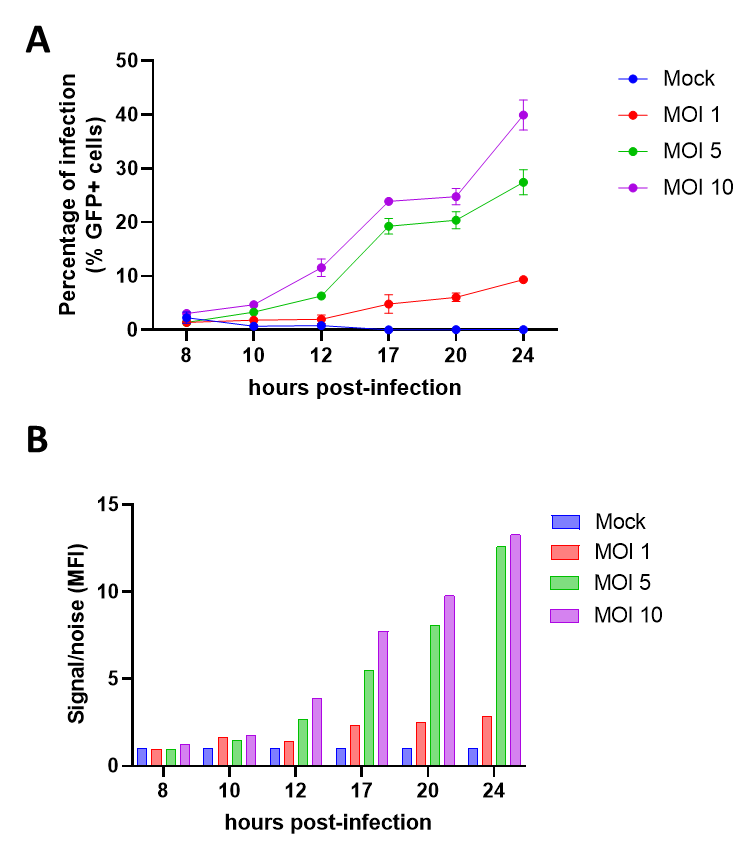
**

**S1 Fig.** Time-course of U2OS cells infection with CHIKV. Cells were infected with either MOI 1, 5 or 10 of 5’GFP-CHIKV-LR, fixed and stained with Hoechst at the indicated time points after infection. Images were acquired in a Cellomics ArrayScan VTI HCS Reader. **(A)** Percentage of GFP+ (infected) cells after each time-point. **(B)** Bar plot showing the signal-to-noise ratio after each time point, i.e, the mean fluorescence intensity (MFI) from infected wells (signal) divided by the MFI of the corresponding mock (noise).
