# Supplementary material for: Mitochondrial protein BNIP3 regulates Chikungunya virus replication in the early stages of infection
Source: PLoS Negl Trop Dis. 2023 Nov 27;17(11):e0010751. doi: 10.1371/journal.pntd.0010751 (PMC10703415; doi:10.1371/journal.pntd.0010751)
Supplement: S2 Fig — (A) Plot shows the number of cells for each gene knockdown in the siRNA-based screen presented in Fig 1A and 1B. Data represents the mean ± SEM of four independent experiments. (DOCX) [file pntd.0010751.s002.docx]

**
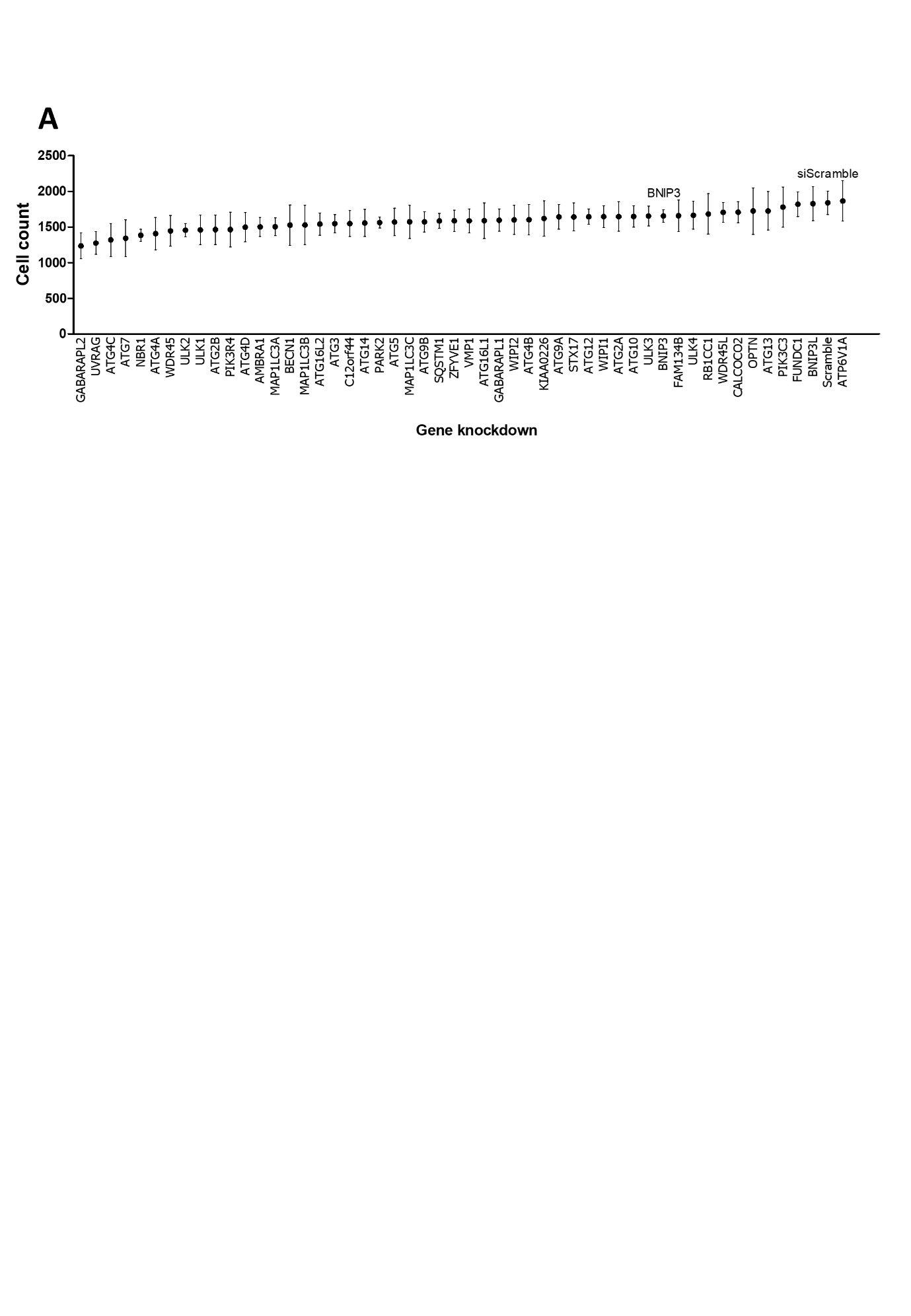
**

**S2 Fig. siRNA transfection does not induce cell toxicity. (A)** Plot shows the number of cells for each gene knockdown in the siRNA-based screen presented in Fig. 1A-B. Data represents the mean ± SEM of four independent experiments.
