# Supplementary material for: Mitochondrial protein BNIP3 regulates Chikungunya virus replication in the early stages of infection
Source: PLoS Negl Trop Dis. 2023 Nov 27;17(11):e0010751. doi: 10.1371/journal.pntd.0010751 (PMC10703415; doi:10.1371/journal.pntd.0010751)
Supplement: S3 Fig — U2OS cells transiently expressing mCherry-BNIP3 were reverse-transfected with either siScramble or siBNIP3 for 16 h. (A) Representative flow cytometry dot plot showing the percentage of mCherry-BNIP3-positive cells. (B) Representative blot and bar plot showing the mCherry protein expression by western blot. (C) Representative fluorescent micrographs taken with a 10X magnification objective of U2OS cells infected with MOI 10 from Fig 1D, prior to collection for flow cytometry. FC denotes for fold change. Data shown represents the mean ± SEM of at least three independent experiments. Student’s test: ** p < 0.01, no symbol implies non-statistically significant. (DOCX) [file pntd.0010751.s003.docx]

**
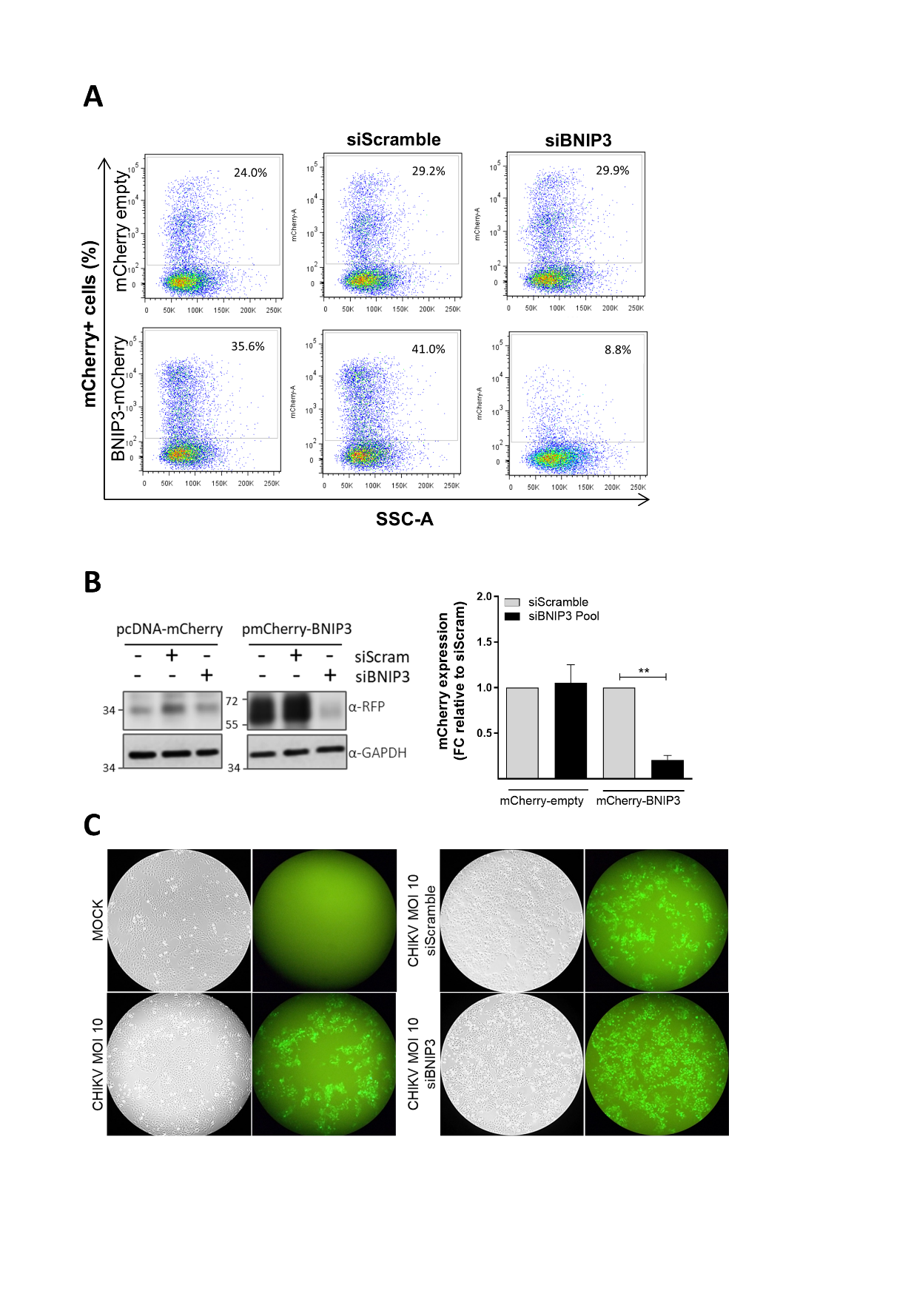
**

**S3 Fig**. **siRNA-mediated knockdown of BNIP3 and validation.** U2OS cells transiently expressing mCherry-BNIP3 were reverse-transfected with either siScramble or siBNIP3 for 16 h. **(A)** Representative flow cytometry dot plot showing the percentage of mCherry-BNIP3-positive cells. **(B)** Representative blot and bar plot showing the mCherry protein expression by western blot. **(C)** Representative fluorescent micrographs taken with a 10X magnification objective of U2OS cells infected with MOI 10 from Fig. 1D, prior to collection for flow cytometry. FC denotes for fold change. Data shown represents the mean ± SEM of at least three independent experiments. Student’s test: ** *p* < 0.01, no symbol implies non-statistically significant.
