# Supplementary material for: Mitochondrial protein BNIP3 regulates Chikungunya virus replication in the early stages of infection
Source: PLoS Negl Trop Dis. 2023 Nov 27;17(11):e0010751. doi: 10.1371/journal.pntd.0010751 (PMC10703415; doi:10.1371/journal.pntd.0010751)
Supplement: S4 Fig — (A) Representative LC3 western blots and (B) determination of the autophagic flux index (LC3-II/LC3-I ratio) in protein lysates from U2OS cells either mock-treated or infected with CHIKV-LR at MOI 10 at the indicated time-points. (C) Representative fluorescence microscopy images and (D) quantification of GFP-WIPI2 puncta in U2OS cells stably expressing GFP-WIPI2 and infected with CHIKV-LR at MOI 10. Cells were analysed at the indicated time-points. (E) Data presented in Fig 2A and 2B was quantified and shown as LC3-II normalised to the housekeeping gene (GAPDH). In all cases, starvation-induced autophagy is used as a positive control (HBSS/EBSS). Baf, Bafilomycin A1. Data shown represent mean ± SEM of at least three independent experiments. Student’s test: *** p < 0.001, ** p < 0.01, no symbol implies non-statistically significant. (DOCX) [file pntd.0010751.s004.docx]

**
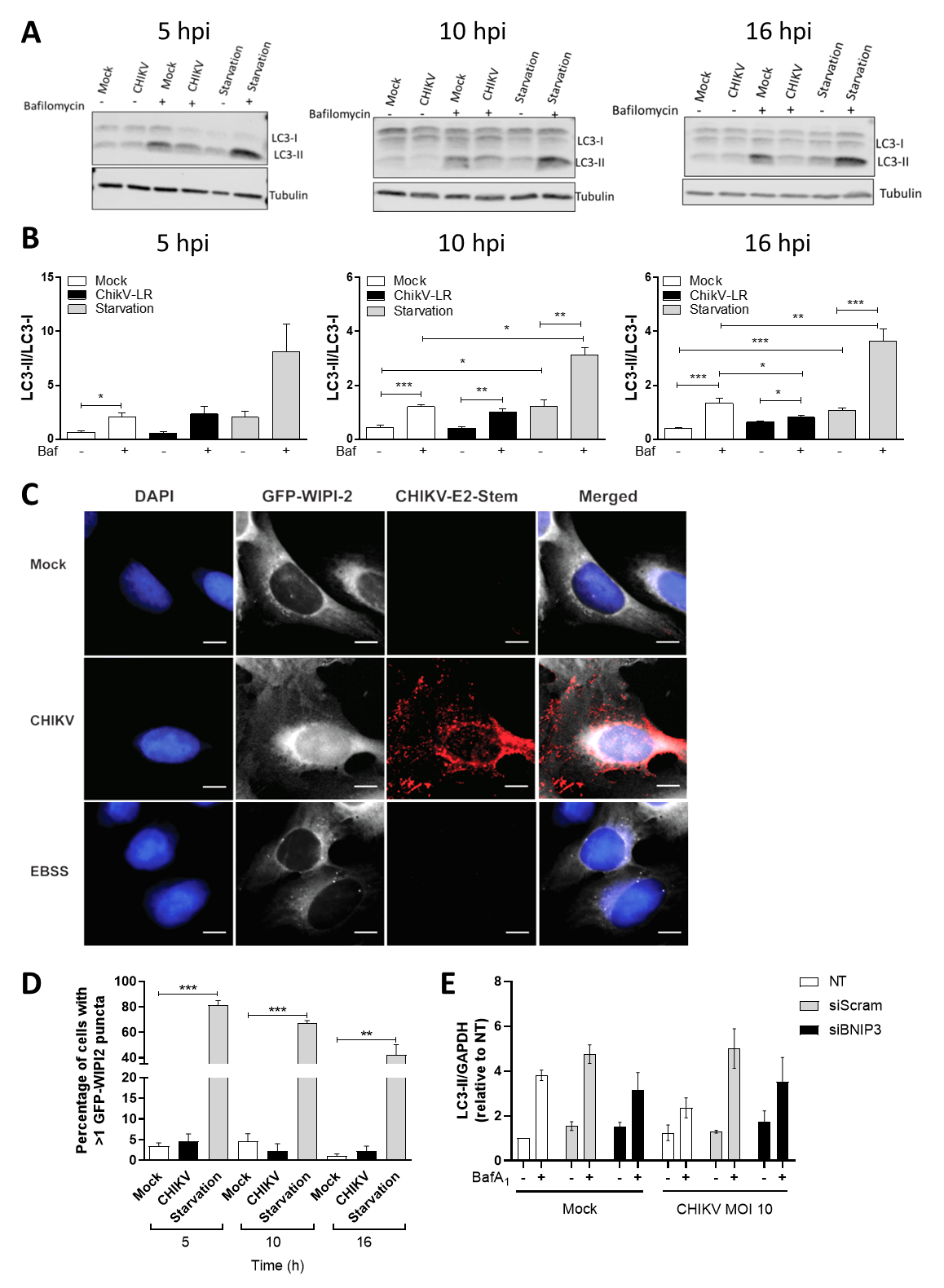
**

**S4 Fig**. **CHIKV does not trigger an autophagic response in U2OS cells.** **(A)** Representative LC3 western blots and **(B)** determination of the autophagic flux index (LC3-II/LC3-I ratio) in protein lysates from U2OS cells either mock-treated or infected with CHIKV-LR at MOI 10 at the indicated time-points. **(C)** Representative fluorescence microscopy images and **(D)** quantification of GFP-WIPI2 puncta in U2OS cells stably expressing GFP-WIPI2 and infected with CHIKV-LR at MOI 10. Cells were analysed at the indicated time-points. **(E)** Data presented in Fig. 2A-B was quantified and shown as LC3-II normalised to the housekeeping gene (GAPDH). In all cases, starvation-induced autophagy is used as a positive control (HBSS/EBSS). Baf, Bafilomycin A_1_. Data shown represent mean ± SEM of at least three independent experiments. Student’s test: *** *p* < 0.001, ** *p* < 0.01, no symbol implies non-statistically significant.
