# Supplementary material for: Mitochondrial protein BNIP3 regulates Chikungunya virus replication in the early stages of infection
Source: PLoS Negl Trop Dis. 2023 Nov 27;17(11):e0010751. doi: 10.1371/journal.pntd.0010751 (PMC10703415; doi:10.1371/journal.pntd.0010751)
Supplement: S5 Fig — (A) Representative flow cytometry histogram of U2OS cells treated with HBSS for 10 h to induce mitochondrial fission and probed using MitoSpy Green. (B-C) U2OS cells were reverse-transfected with siBNIP3 or siScramble for 48 h and infected with CHIKV-LR for 10 h at the indicated MOI and stained with mitochondrial probes. Bar plots show (B) the total mitochondrial mass (MitoSpy Green FM), or (C) the mass of polarized mitochondria (MitoSpy Red CMXRos), relative to mock-treated cells. (D) Representative flow cytometry dot plot and quantification of U2OS cells treated with menadione at a concentration of 100 μM for 10 h and stained using Annexin V and FVD. NT denotes for non-transfected. FC denotes for fold change. Data shown represent mean ± SEM of at least three independent experiments. Student’s test: no symbol implies non-statistically significant. (DOCX) [file pntd.0010751.s005.docx]

**
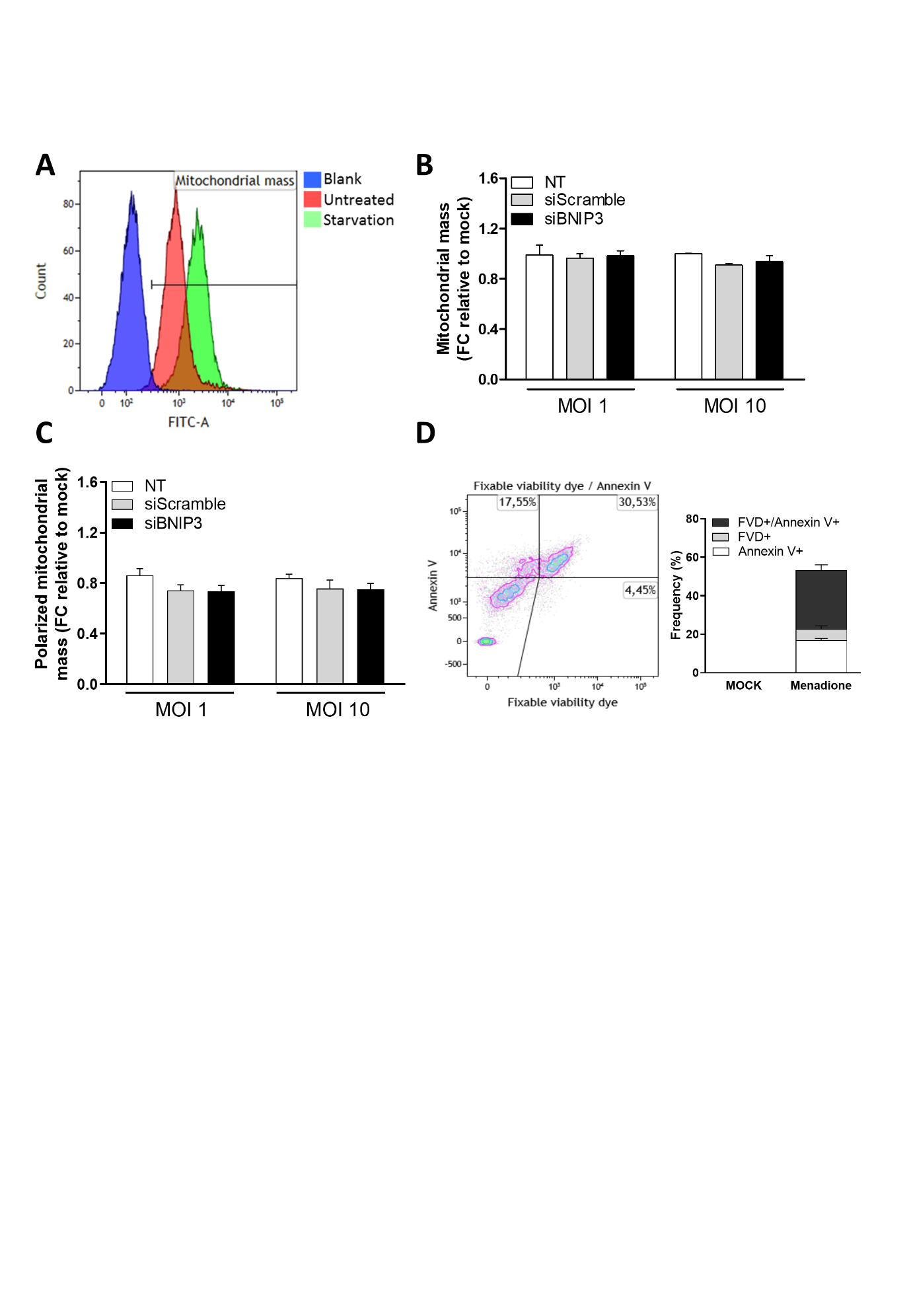
 S5 Fig. Determination of mitochondrial mass and cell death in U2OS cells by flow cytometry. (A)** Representative flow cytometry histogram of U2OS cells treated with HBSS for 10 h to induce mitochondrial fission and probed using MitoSpy Green. **(B-C)** U2OS cells were reverse-transfected with siBNIP3 or siScramble for 48 h and infected with CHIKV-LR for 10 h at the indicated MOI and stained with mitochondrial probes. Bar plots show **(B)** the total mitochondrial mass (MitoSpy Green FM), or **(C)** the mass of polarized mitochondria (MitoSpy Red CMXRos), relative to mock-treated cells. **(D)** Representative flow cytometry dot plot and quantification of U2OS cells treated with menadione at a concentration of 100 µM for 10 h and stained using Annexin V and FVD. NT denotes for non-transfected. FC denotes for fold change. Data shown represent mean ± SEM of at least three independent experiments. Student’s test: no symbol implies non-statistically significant.
