# Supplementary material for: Mitochondrial protein BNIP3 regulates Chikungunya virus replication in the early stages of infection
Source: PLoS Negl Trop Dis. 2023 Nov 27;17(11):e0010751. doi: 10.1371/journal.pntd.0010751 (PMC10703415; doi:10.1371/journal.pntd.0010751)
Supplement: S6 Fig — U2OS cells were reverse-transfected with siBNIP3 or siScramble for 48 h, and infected with CHIKV-LR for 10 h. (A) Bar plot showing the intracellular levels of E1 and nsP1 RNA (expressed as RNA copies/ml of cell lysate) in cells transfected with siScramble and infected at the indicated MOI and time-points. (B) Bar plot showing the titration of infectious CHIKV particles per ml of supernatant (PFU/ml, determined by plaque assay) after 16 hpi and expressed as relative to NT cells. NT denotes for non-transfected. FC denotes for fold change. Data shown represent mean ± SEM of three independent experiments. Student’s test: *** p < 0.001, ** p < 0.01, * p < 0.05, no symbol implies non-statistically significant. (DOCX) [file pntd.0010751.s006.docx]

**
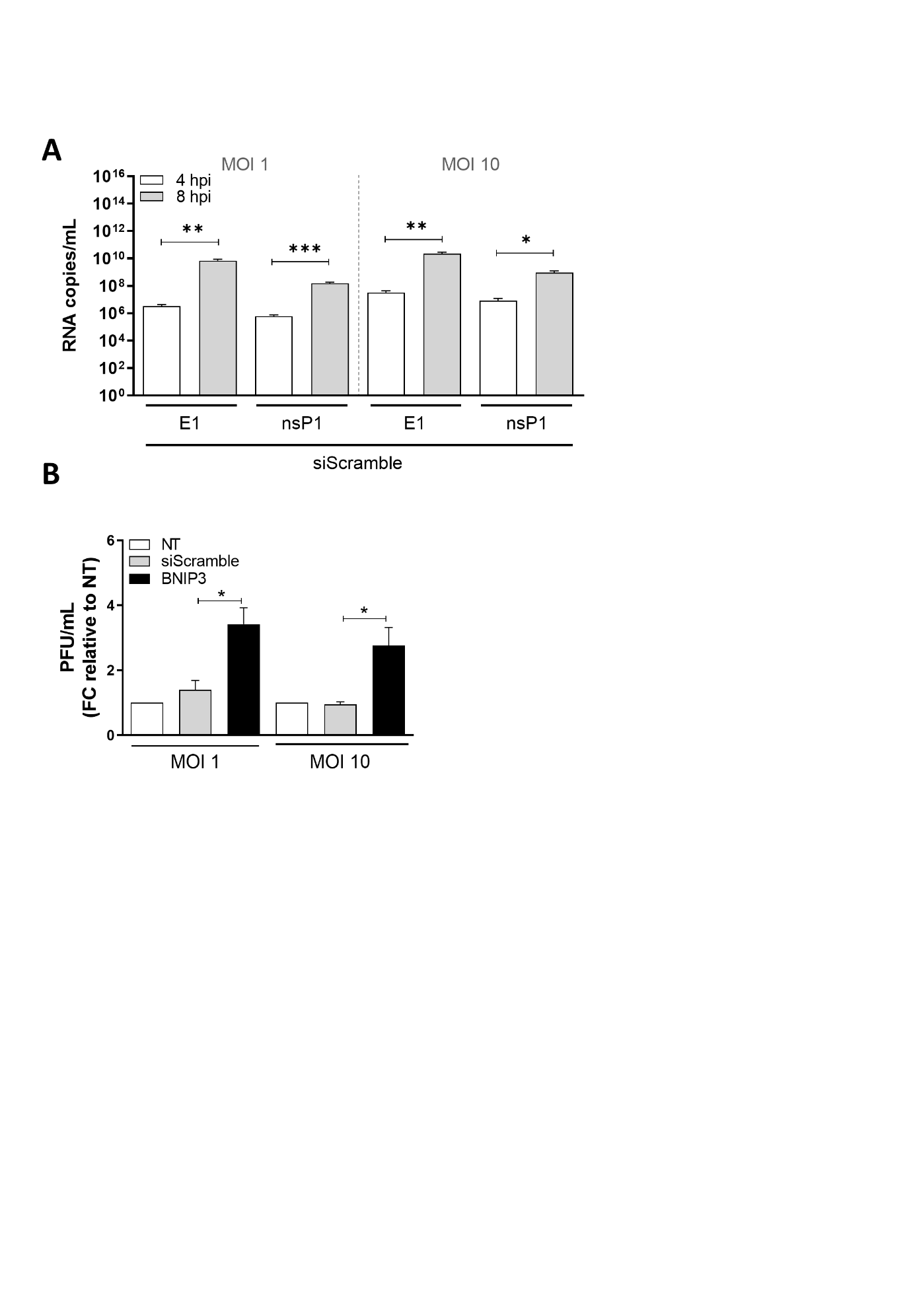
**

**S6 Fig. CHIKV RNA levels and virus titres are enhanced by BNIP3 silencing.** U2OS cells were reverse-transfected with siBNIP3 or siScramble for 48 h, and infected with CHIKV-LR for 10 h. **(A)** Bar plot showing the intracellular levels of E1 and nsP1 RNA (expressed as RNA copies/ml of cell lysate) in cells transfected with siScramble and infected at the indicated MOI and time-points. **(B)** Bar plot showing the titration of infectious CHIKV particles per ml of supernatant (PFU/ml, determined by plaque assay) after 16 hpi and expressed as relative to NT cells. NT denotes for non-transfected. FC denotes for fold change. Data shown represent mean ± SEM of three independent experiments. Student’s test: *** *p* < 0.001, ** *p* < 0.01, * *p* < 0.05, no symbol implies non-statistically significant.
