# Supplementary material for: Mitochondrial protein BNIP3 regulates Chikungunya virus replication in the early stages of infection
Source: PLoS Negl Trop Dis. 2023 Nov 27;17(11):e0010751. doi: 10.1371/journal.pntd.0010751 (PMC10703415; doi:10.1371/journal.pntd.0010751)
Supplement: S1 Table — (DOCX) [file pntd.0010751.s007.docx]

| **Gene Symbol** | **Gene ID** | **Gene Accession** |
| --- | --- | --- |
| ULK1 | 8408 | NM_003565 |
| ULK2 | 9706 | NM_014683 |
| ULK3 | 25989 | NM_015518 |
| ULK4 | 54986 | XM_929989 |
| ATG2A | 23130 | NM_015104 |
| ATG2B | 55102 | NM_018036 |
| ATG3 | 64422 | NM_022488 |
| ATG4A | 115201 | NM_178270 |
| ATG4B | 23192 | NM_013325 |
| ATG4C | 84938 | NM_178221 |
| ATG4D | 84971 | NM_032885 |
| ATG5 | 9474 | NM_004849 |
| BECN1 | 8678 | NM_003766 |
| ATG7 | 10533 | NM_006395 |
| MAP1LC3A | 84557 | NM_181509 |
| MAP1LC3B | 81631 | NM_022818 |
| MAP1LC3C | 440738 | NM_001004343 |
| GABARAPL1 | 23710 | NM_031412 |
| GABARAPL2 | 11345 | NM_007285 |
| ATG9A | 79065 | NM_024085 |
| ATG9B | 285973 | NM_173681 |
| ATG10 | 83734 | NM_031482 |
| ATG12 | 9140 | NM_004707 |
| ATG13 | 9776 | NM_014741 |
| ATG14 | 22863 | NM_014924 |
| ATG16L1 | 55054 | NM_198890 |
| ATG16L2 | 89849 | NM_033388 |
| RB1CC1 | 9821 | NM_014781 |
| WIPI1 | 55062 | NM_017983 |
| WIPI2 | 26100 | NM_001033520 |
| WDR45B | 56270 | NM_019613 |
| WDR45 | 11152 | NM_001029896 |
| ATG101 | 60673 | NM_021934 |
| ZFYVE1 | 53349 | NM_178441 |
| AMBRA1 | 55626 | NM_017749 |
| VMP1 | 81671 | NM_030938 |
| SQSTM1 | 8878 | NM_003900 |
| NBR1 | 4077 | NM_005899 |
| CALCOCO2 | 10241 | NM_005831 |
| OPTN | 10133 | NM_021980 |
| PIK3C3 | 5289 | NM_002647 |
| PIK3R4 | 30849 | NM_014602 |
| UVRAG | 7405 | NM_003369 |
| KIAA0226 | 9711 | XM_032901 |
| STX17 | 55014 | NM_017919 |
| FAM134B | 54463 | NM_019000 |
| PARK2 | 5071 | NM_013988 |
| BNIP3L | 665 | NM_004331 |
| FUNDC1 | 139341 | NM_173794 |
| BNIP3 | 664 | NM_004052 |
| ATP6V1A | 523 | NM_001690 |

**S1 Table.** Genes targeted by the customised siRNA library and gene accession numbers.
