# Supplementary material for: Mitochondrial protein BNIP3 regulates Chikungunya virus replication in the early stages of infection
Source: PLoS Negl Trop Dis. 2023 Nov 27;17(11):e0010751. doi: 10.1371/journal.pntd.0010751 (PMC10703415; doi:10.1371/journal.pntd.0010751)
Supplement: S2 Table — (DOCX) [file pntd.0010751.s008.docx]

| **Cell line** | **MOI** | **Time post-infection (h)** | **Percentage of infection** | | | **Relative to NT** | **SEM** | **N** |
| --- | --- | --- | --- | --- | --- | --- | --- | --- |
|  |  |  | **NT** | **siScramble** | **siBNIP3** |  |  |  |
| U2OS | 1 | 10 | 4.41 | 5.50 | 9.31 | 2.11 | 0.16 | 4 |
|  |  |  | 2.73 | 2.58 | 5.93 | 2.17 |  |  |
|  |  |  | 3.69 | 3.95 | 5.25 | 1.42 |  |  |
|  |  |  | 2.63 | 2.55 | 6.26 | 2.38 |  |  |
|  | 10 |  | 14.60 | 13.53 | 30.32 | 2.08 | 0.18 | 4 |
|  |  |  | 17.93 | 17.22 | 34.50 | 1.92 |  |  |
|  |  |  | 14.99 | 11.74 | 18.92 | 1.26 |  |  |
|  |  |  | 11.94 | 13.18 | 28.34 | 2.37 |  |  |

**S2 Table**. Raw data belonging to Figure 1D.
