# Supplementary material for: Mitochondrial protein BNIP3 regulates Chikungunya virus replication in the early stages of infection
Source: PLoS Negl Trop Dis. 2023 Nov 27;17(11):e0010751. doi: 10.1371/journal.pntd.0010751 (PMC10703415; doi:10.1371/journal.pntd.0010751)
Supplement: S3 Table — (DOCX) [file pntd.0010751.s009.docx]

| **Cell line** | **MOI** | **Time post- infection (h)** | **Percentage of infection** | | **Relative to siScramb**l**e** | **SD** | **N** |
| --- | --- | --- | --- | --- | --- | --- | --- |
|  |  |  | **siScramble** | **siBNIP3** |  |  |  |
| MRC-5 | 1 | 10 | 24.11 | 36.09 | 1.50 | 0.13 | 4 |
|  | 1 | 10 | 25.02 | 40.66 | 1.63 |  |  |
|  | 1 | 10 | 18.73 | 33.32 | 1.78 |  |  |
|  | 1 | 10 | 22.94 | 39.93 | 1.74 |  |  |
| HuH7 | 5 | 10 | 42.82 | 44.61 | 1.04 | 0.07 | 3 |
|  | 5 | 10 | 39.52 | 41.58 | 1.05 |  |  |
|  | 5 | 10 | 40.89 | 37.89 | 0.93 |  |  |
| HeLa | 5 | 10 | 9.16 | 8.23 | 0.90 | 0.13 | 3 |
|  | 5 | 10 | 9.18 | 8.64 | 0.94 |  |  |
|  | 5 | 10 | 6.76 | 7.74 | 1.14 |  |  |

**S3 Table**. Raw data belonging to Figure 1E.
