# Supplementary material for: Mitochondrial protein BNIP3 regulates Chikungunya virus replication in the early stages of infection
Source: PLoS Negl Trop Dis. 2023 Nov 27;17(11):e0010751. doi: 10.1371/journal.pntd.0010751 (PMC10703415; doi:10.1371/journal.pntd.0010751)
Supplement: S4 Table — (DOCX) [file pntd.0010751.s010.docx]

| **Percentage of infection** | | | | | **Relative to siScramble** | | | **N** |
| --- | --- | --- | --- | --- | --- | --- | --- | --- |
| **NT** | **siScramble** | **siBNIP3** | **siATG7** | **siBNIP3 + siATG7** | **siBNIP3** | **siATG7** | **siATG7 + siBNIP3** |  |
| 22 | 12 | 28.3 | 19.3 | 25.6 | 2.36 | 1.61 | 2.13 | 5 |
| 32.9 | 21 | 39.3 | 29.3 | 40.1 | 1.87 | 1.40 | 1.91 |  |
| 20.35 | 15.44 | 32.51 | 24.43 | 35.42 | 2.11 | 1.58 | 2.29 |  |
| 27.26 | 22.53 | 51.55 | 38.23 | 55.05 | 2.29 | 1.70 | 2.44 |  |
| 17.52 | 11.58 | 29.37 | 22.45 | 30.75 | 2.13 | 1.63 | 2.66 |  |
| 21.12 | 15.22 | 43.51 | 24.52 | 34.2 | 2.86 | 1.61 | 2.25 |  |

**S4 Table.** Raw data belonging to Figure 2D. U2OS cells were infected with MOI 10 of CHIKV-LR, data corresponds to 10 hpi.
