# Supplementary material for: Mitochondrial protein BNIP3 regulates Chikungunya virus replication in the early stages of infection
Source: PLoS Negl Trop Dis. 2023 Nov 27;17(11):e0010751. doi: 10.1371/journal.pntd.0010751 (PMC10703415; doi:10.1371/journal.pntd.0010751)
Supplement: Attachment — Submitted filename: Cover letter PNTD_afterappeal.pdf [file pntd.0010751.s012.pdf]

August, 15th, 2023

Editor-in-chief
PLoS Neglected Tropical Diseases

Dear Editor,

Thank you for approving the appeal request and herewith we re-submit our original research manuscript entitled: '**Mitochondrial protein BNIP3 regulates chikungunya virus replication in the early stages of infection**' by Echavarria-Consuegra SL, Dinesh-Kumar N, M. van der Laan, Mauthe M, Van de Pol D, Reggiori F and Smit JM for publication in *PLoS Neglected Tropical Diseases.*

Based on the previous reviewing period we have now added more 'raw' data in the text and as supplementary information to the manuscript. These can be found in Table S2-4.

After the approval of the appeal, we also revised the manuscript's title and revised the discussion section as requested. Furthermore, we now explicitly describe how BNIP3 regulates autophagy including the role of mTOR and ULK1. However, based on our findings that the ATG machinery was not deemed significant and UKL1 functions upstream of it, it is unlikely that UKL1 plays a crucial role. Therefore, we do not see the added value of testing UKL1 and we do not consider this as a limitation of this manuscript.

During the initial resubmission of the manuscript, I had a discussion with the journal administration regarding the addition of Marleen van der Laan as an author to the manuscript. They informed me that I can add her during the production stages if I provide a written response from all authors agreeing to her inclusion. I have already uploaded this agreement file along with the manuscript files.

We hope that with these changes the manuscript is acceptable for publication in PloS NTD.

On behalf of all authors,

Sincerely,

Jolanda M. Smit
