# Supplementary material for: Mitochondrial protein BNIP3 regulates Chikungunya virus replication in the early stages of infection
Source: PLoS Negl Trop Dis. 2023 Nov 27;17(11):e0010751. doi: 10.1371/journal.pntd.0010751 (PMC10703415; doi:10.1371/journal.pntd.0010751)
Supplement: Attachment — Submitted filename: BNIP3_rebuttal letter V2[29].docx [file pntd.0010751.s011.docx]

Echavarria-Consuegra et al

***Reviewer's Responses to Questions***

***Summary and General Comments***

*Reviewer #1: Flawless work from all points of view: Methodological rigor, syntax, clarity in the presentation, and presentation of the results. Considerable discussions with comparisons with the literature.*

*Few minor points to address:*

1. *Please describe how the experimental conditions (MOI, hpi) of viral infection were established. Are they determined by preliminary experiments or inferred from literature?*

We indeed indicated on page 16, line 388 that we had selected optimal experimental conditions in terms of multiplicity of infection (MOI) and time-point after infection to perform the siRNA screen. We acknowledge, however, that we did not describe this in much detail. To follow this reviewer’s suggestion, this section of the manuscript has been expanded, and we have included a new supplementary figure (new Figure S1A and S1B) showing the results of the experiments that were performed prior to the siRNA screen and that informed us on the selection of the infection conditions of the experiments that followed. In brief, the new Figures S1A and S1B show that infection of U2OS cells with MOI 10 of CHIKV results in a GFP signal that can be unambiguously distinguished from the mock after ~16 hours post infection (hpi). In addition, the percentage of infection at this time-point was 23.9%, enabling identification of protein knockdowns that alter infection above or below that threshold.

2. *In autophagic flux experiments, the expression levels of LC3-II in presence and absence of the autophagic inhibitor/stimulator should be preferred instead of LC3-II/LC3-I ratio.*

The ratio between autophagosome-associated LC3-II to cytoplasmic LC3-I in the presence and absence of the lysosomal pH inhibitor bafilomycin A1, known as autophagy flux index, is preferred as a marker of autophagy flux, as omitting the quantification of the levels of LC3-I in favour of normalised LC3-II may not provide a full picture of the dynamics of the autophagy response (Klionsky et al., 2016). Nonetheless, we re-analysed the data presented in Figure 2A and 2B as suggested by this reviewer, namely by quantifying LC3-II in the presence and absence of bafilomycin A1 and normalising against the housekeeping gene (GAPDH). The results of the new analysis, presented in the new Figure S4E, fully confirm our original interpretations, that is, autophagy flux during CHIKV infection occurs independently of BNIP3 expression. We now explicitly disclose this in page 21, line 541-543 of the manuscript.

3. *To avoid off target effects, is it possible to evaluate the autophagic flux in infected cells treated with other inhibitors (such as chloroquine) and activators (rapamycin)?*

Although chemical autophagy inhibitors and inducers have facilitated the investigation of autophagy, most of these drugs are not entirely specific to their targets, and therefore, genetic intervention is a favoured approach to study autophagy (Y. Yang et al., 2013). For example, in the case of rapamycin, both immunosuppressive and antiproliferative effects have been well established (Li et al., 2014). Similarly, cloroquine has multiple side effects both *in vitro* and *in vivo*, including immunomodulatory effects (Savarino et al., 2003) and the disorganization of the Golgi and endo-lysosomal system (Mauthe et al., 2018). Due to these multiple modes of action, cloroquine has been studied as a potential antiviral for several viruses of the *Flaviviridae*, *Retroviridae* and *Coronaviridae* families (Lamballerie et al., 2008; Savarino et al., 2003). For all of the above, we selected for our study CRISPR-Cas9 knockout-cells for the autophagy initiation factor ATG7 and knockdown of individual autophagy genes, to produce a more specific manipulation of autophagy.

4. *Similarly, to avoid cell-specific effects, representative experiments (for example, the validation of BNIP3-mediated CHIKV infection potential independently of autophagy) should be performed in other CHIKV permissive cell lines (such as HEK293T, HepG2, Hela)*

Based on the comments of the reviewer we assessed the effect of BNIP3 in other cell lines. The results are added to the manuscript as Fig. 1E. In short, and in line with the results in U2OS cells, depletion of BNIP3 was found to increase the number of infected cells following infection in the human fibroblast cell line MRC-5. Next to MRC-5, we also assessed the effect in HeLa and HuH-7 cells were no enhancement of infection was seen. We elaborate on these results in the manuscript in page 19, line 469-476.

*Reviewer #2: The authors that examined the BNIP3 gene function in CHIKV replication. They are using siRNA approach for screening ATG-related gene and BNIP3 gene with viral infection. Then, they found that BNIP3 can increase CHIKV infection, but did not induce cell death and autophagy. Some comments as following:*

1. *If BNIP3 is not correlated to cell death or autophagy process, but why have some papers was demonstrated them. Here, the authors should provide more strongly evidences on this.*

We want to emphasise that the data presented in our manuscript does not contradict nor deny the function of BNIP3 in cell death and autophagy in other pathogenic models such as hypoxia and cardiac arrest; which have been thoroughly studied by other authors. This study is centralized around the role of BNIP3 in the context of CHIKV infection. Our findings provide new ways in which the molecular networks of BNIP3 can be interpreted. In order to clarify this, we changed the phrasing in the discussion to match our reasoning (page 30, line 791-795).

We would also like to point out that at almost every stage of our research the findings of one experiment were corroborated and contrasted using two or more different methodologies or techniques.

2. *The authors may just said BNIP3 can regulate the CHIKV replication during virus replication cycle, but should have more viral protein expression profile.*

Although we do not fully understand the reasoning behind this comment, we did analyse and quantified the expression of both non-structural (nsP2) and structural proteins (E1, E2, capsid) of CHIKV under conditions of BNIP3 depletion by western blot. These results are shown in Figure 5B and 5C.

3. *In figure 3A, knock-downed the BNIP3 it seems to enhance the mitochondria broken-down in Mock+siBNIP3 and CHIKV+ siBNIP3 groups.*

In Figure 3A we used cells stably expressing Cox-8 fused to mCherry and GFP to monitor the delivery of mitochondria to autophagosomes and their subsequent degradation in mitolysosomes by fluorescence microscopy. Given that interpretation of this type of imaging data can be highly subjective, we quantified the red-only puncta, indicative of mitolysosomes in 50 cells of 3 independent biological repeats; for a more objective analysis of our observations. We disclosed the method of quantification in the materials and methods section (page 14, line 334), and have now also explicitly stated this in the legend of Figure 3. The results of the mitolysosomes quantifications are presented in the manuscript in Figure 3B, and are sufficient evidence that mitochondrial delivery to autolysosomes is not enhanced nor inhibited by BNIP3 expression in CHIKV-infected cells. In agreement with this data, no significant differences were observed in the total mitochondrial mass in cells depleted of BNIP3, as shown in Fig. S4B. As a final remark on this comment and in order to accompany this data set, we have included a new figure (Figure 3C), showing the quantification of the percentage of infected cells in these experiments. The result presented in this figure is now described in the text in page 22, line 583-584.

4. *Autophagy process is a flow process should have some signaling pathways induction, such as mTOR/ULK1 or AKT/mTOR/ULK1 did not just said from gene expression level, which still have some protein modification such as protein phosphorylation.*

The siRNA screen presented in the first section of our study was performed as an initial, unbiased approach to identify autophagy factors that are prominent modulators of CHIKV replication cycle. We used a siRNA library (Table S1) targeting the known ATG proteome; autophagy receptors; and included combinations of siRNAs targeting proteins that make part of the same complex/step in the process of autophagy (page 15, line 377). To the best of our knowledge, we have covered all the steps of the pathway. Our observations indicate that the different isoforms of ULK proteins, have potential antiviral effect against CHIKV (page 17, line 422 to 428). Likewise, in agreement with other authors, we identified that the autophagy pathway proteins like ATG7, have a negative impact on CHIKV infection (Judith et al., 2013; L. Yang et al., 2020). However, we decided to focus our research efforts into the validation of BNIP3, a protein that had not been reported before in connection to the infection by alphaviruses and that also showed the highest upregulation of CHIKV percentage of infection when depleted. It was, therefore, out of the scope of this study to investigate ULK proteins and its upstream signalling regulators (such as mTOR or AKT).

5. *If possible the authors should provide a cartoon to summary the results for more ease to check the point.*

We found this to be a valuable suggestion, therefore, we added a new schematic representation of the involvement of BNIP3 in CHIKV replication cycle which is now the new Figure 7.

**References**

Judith, D., Mostowy, S., Bourai, M., Gangneux, N., Lelek, M., Lucas-Hourani, M., Cayet, N., Jacob, Y., Prévost, M.-C. C., Pierre, P., Tangy, F., Zimmer, C., Vidalain, P.-O. O., Couderc, T., & Lecuit, M. (2013). Species-specific impact of the autophagy machinery on Chikungunya virus infection. *EMBO Reports*, *14*(6), 534–544. https://doi.org/10.1038/embor.2013.51

Klionsky, D. J., Abdelmohsen, K., Abe, A., Abedin, M. J., Abeliovich, H., Acevedo Arozena, A., Adachi, H., Adams, C. M., Adams, P. D., Adeli, K., Adhihetty, P. J., Adler, S. G., Agam, G., Agarwal, R., Aghi, M. K., Agnello, M., Agostinis, P., Aguilar, P. v., Aguirre-Ghiso, J., … Zughaier, S. M. (2016). Guidelines for the use and interpretation of assays for monitoring autophagy (3rd edition). *Autophagy*, *12*(1), 1–222. https://doi.org/10.1080/15548627.2015.1100356

Lamballerie, X. de, Boisson, V., Reynier, J.-C., Enault, S., Charrel, R. N., Flahault, A., Roques, P., & Grand, R. le. (2008). On Chikungunya Acute Infection and Chloroquine Treatment. *Vector-Borne and Zoonotic Diseases*, *8*(6), 837–840. https://doi.org/10.1089/vbz.2008.0049

Li, J., Kim, S. G., & Blenis, J. (2014). Rapamycin: One Drug, Many Effects. *Cell Metabolism*, *19*(3), 373–379. https://doi.org/10.1016/j.cmet.2014.01.001

Mauthe, M., Orhon, I., Rocchi, C., Zhou, X., Luhr, M., Hijlkema, K. J., Coppes, R. P., Engedal, N., Mari, M., & Reggiori, F. (2018). Chloroquine inhibits autophagic flux by decreasing autophagosome-lysosome fusion. *Autophagy*, *14*(8), 1435. https://doi.org/10.1080/15548627.2018.1474314

Savarino, A., Boelaert, J. R., Cassone, A., Majori, G., & Cauda, R. (2003). Effects of chloroquine on viral infections: an old drug against today’s diseases. *The Lancet Infectious Diseases*, *3*(11), 722–727. https://doi.org/10.1016/S1473-3099(03)00806-5

Yang, L., Geng, T., Yang, G., Ma, J., Wang, L., Ketkar, H., Yang, D., Lin, T., Hwang, J., Zhu, S., Wang, Y., Dai, J., You, F., Cheng, G., Vella, A. T., Flavell, R. A., Fikrig, E., & Wang, P. (2020). Macrophage scavenger receptor 1 controls Chikungunya virus infection through autophagy in mice. *Communications Biology 2020 3:1*, *3*(1), 1–11. https://doi.org/10.1038/s42003-020-01285-6

Yang, Y., Hu, L., Zheng, H., Mao, C., Hu, W., Xiong, K., Wang, F., & Liu, C. (2013). Application and interpretation of current autophagy inhibitors and activators. *Acta Pharmacologica Sinica*, *34*(5), 625–635. https://doi.org/10.1038/aps.2013.5
